# Can we improve transthoracic echocardiography training in non-cardiologist residents? Experience of two training programs in the intensive care unit

**DOI:** 10.1186/s13613-016-0150-8

**Published:** 2016-05-17

**Authors:** Vincent Labbé, Stéphane Ederhy, Blandine Pasquet, Romain Miguel-Montanes, Cédric Rafat, David Hajage, Stéphane Gaudry, Didier Dreyfuss, Ariel Cohen, Muriel Fartoukh, Jean-Damien Ricard

**Affiliations:** Unité de Réanimation médico-chirurgicale, Pôle Thorax Voies Aériennes, Groupe hospitalier des Hôpitaux Universitaires de l’Est Parisien, Hôpital Tenon, Assistance Publique-Hôpitaux de Paris, 4 rue de la Chine, 75020 Paris, France; Service de Cardiologie, Groupe hospitalier des Hôpitaux Universitaires de l’Est Parisien, Hôpital Saint Antoine, Assistance Publique-Hôpitaux de Paris, Paris, France; Département d’épidémiologie et de recherche clinique, Hôpital Louis Mourier, Assistance Publique-Hôpitaux de Paris, Colombes, France; Service de Réanimation Médico-Chirurgicale, Hôpital Louis Mourier, Colombes, Assistance Publique-Hôpitaux de Paris, Paris, France; Intensive Care Department, Geneva University Hospitals, Geneva, Switzerland; Urgences Néphrologiques et Transplantation Rénale, Groupe hospitalier des Hôpitaux Universitaires de l’Est Parisien, Hôpital Tenon, Assistance Publique-Hôpitaux de Paris, Paris, France; Centre de Pharmacoépidémiologie de l’Assistance Publique-Hôpitaux de Paris (Cephepi), Paris, France; UMR 1123 ECEVE, Sorbonne Paris Cité, Université Paris Diderot, Paris, France; INSERM, CIC 1425-EC, Paris, France; INSERM, UMR 1123 ECEVE, Paris, France; Institut National de la Santé et de la Recherche Médicale (INSERM), IAME, UMR 1137, 75018 Paris, France; IAME 1137, Université Paris Diderot, 75018 Paris, France; Sorbonne Universités, Université Pierre et Marie Curie, Paris 06, Paris, France

**Keywords:** Transthoracic echocardiography, Curriculum, Intensive care, Academic training

## Abstract

**Background:**

To evaluate the diagnostic performances of two training programs for residents with no prior ultrasound experience to reach competences in extended basic critical care transthoracic echocardiography (CCE) including Doppler capabilities.

**Methods:**

This is a prospective observational study in two intensive care units of teaching hospitals. Group I (five residents) completed a short training program (4-h theory; 3-h practical); group II (six residents) completed a longer training program (6-h theory; 12-h practical). The residents and an expert examined all patients who required a transthoracic echocardiography. Their agreement studied by Cohen’s κ coefficient, concordance coefficient correlation (CCC) and Bland–Altman plots was used as an indicator of program effectiveness.

**Results:**

Group I performed 136 CCEs (mean/resident 27; range 22–32; 65 in ventilated patients) in 115 patients (62 men; 64 ± 18 years; Simplified Acute Physiologic Score [SAPS] II 37 ± 18). Group II performed 158 CCEs (mean/resident 26; range 21–31; 65 in ventilated patients) in 108 patients (64 men; 58 ± 17 years; SAPS II 42 ± 22). Both groups adequately assessed left ventricular (LV) systolic function (κ 0.75, 95 % confidence interval [CI] 0.64–0.86; κ 0.77, 95 % CI 0.66–0.88, respectively) and pericardial effusion (κ 0.83, 95 % CI 0.67–0.99; κ 0.76, 95 % CI 0.60–0.93, respectively). Group II appraised severe right ventricular dilatation and significant left-sided valve disease with good to very good agreement (κ 0.80, 95 % CI 0.56–0.96; κ 0.79, 95 % CI 0.66–0.93, respectively). Regarding left ventricular ejection fraction, E/A ratio, E/e′ ratio and aortic peak velocity assessed by group II, CCCs were all >0.70 and the bias (mean difference) ±SD on Bland–Altman analysis was 1.3 ± 8.8 %, 0 ± 0.3, 0.4 ± 2.2 and 0.1 ± 0.4 m/s, respectively. Detection of paradoxical septum (κ 0.65, 95 % CI 0.37–0.93), of heterogeneous LV contraction (κ 0.49, 95 % CI 0.33–0.65) and of respiratory variation of the inferior vena cava (κ 0.27, 95 % CI 0.09–0.45), as well as stroke volume measurement (CCC 0.65, 95 % CI 0.54–0.74; bias ± SD −1.4 ± 4.7 cm), was appraised by group II with moderate agreement requiring probably more comprehensive training.

**Conclusions:**

Although a training program blending 6-h theory and 12-h practical may be adapted to achieve some essential competences, it seems to be insufficiently to perform a complete extended basic critical care transthoracic echocardiography including Doppler capabilities.

**Electronic supplementary material:**

The online version of this article (doi:10.1186/s13613-016-0150-8) contains supplementary material, which is available to authorized users.

## Background

Critical care transthoracic echocardiography (CCE) with Doppler is increasingly used at the bedside of intensive care unit (ICU) patients to establish diagnoses and guide the management of those with cardiopulmonary compromise [1]. Though the availability of echocardiography machines with high-quality two-dimensional (2D) imaging and Doppler capabilities is growing in ICUs, its utilization requires around-the-clock trained operators [1]. While basic CCE is performed as a goal-directed qualitative examination using 2D imaging to answer a limited number of clinical questions, a comprehensive hemodynamic assessment requires the use of Doppler echocardiography capabilities [1]. Therefore, there is an urgent need for education in this specific field of competence that is mandatory in every ICU physician’s curriculum [2]. A major focus of the university ICUs is to promote residents’ education in critical care echocardiography, regardless of their primary specialty. Therefore, it appears necessary to establish a training program for non-cardiologist residents with no prior experience in ultrasound.

The ability to master spectral Doppler and tissue Doppler imaging (TDI) indices after a limited training program has not yet been tested [3]. In fact, the few studies that have attempted to evaluate focused training (ranging from 6 to 15 h) were all limited to basic echocardiography using handheld miniaturized systems without Doppler in ICU settings [4–7]. In order to be reproducible, a training program dedicated to residents with a 6-month rotation period has to be fast and efficient, to provide them with time to carry out their mission of care and acquire other important skills. Therefore, we hypothesized that previously focused training programs dedicated to non-cardiologist residents could be improved to perform extended basic CCE, including common parameters derived from spectral Doppler and TDI. Our second hypothesis was that a long training program would be more efficient than a short training program.

So, this study was designed to evaluate the feasibilities and diagnostic performances of two different focused training programs (a short and a long program) for residents without any previous knowledge in ultrasound for performing extended basic CCE with Doppler capabilities.

## Methods

### Study design

We performed a prospective, observational, educational study comparing two focused training programs to acquire extended basic CCE skills. Our study was considered to be part of routine clinical practice by the appropriate ethics committee (Commission Ethique de la Société de Réanimation de Langue Française, SRLF, Number 14-29), so no informed consent was required from the patients or their relatives. If patients were awake or if their relatives were present, information was given on the training of the residents.

### Training programs

Two distinct training programs were conducted with non-cardiologist residents with no prior experience in ultrasound. Both training programs were conducted by V.L., a cardiologist expert with board certification in echocardiography and level 3 competence according to the American Society of Echocardiography Standards [8].

#### Training program I

Five residents (group I) participated in training program I, which was conducted within the ICU of the University teaching hospital of Colombes (France) between November 2010 and November 2011. It involved 2 months of theoretical and practical appraisal. The theoretical program (detailed in Additional file 1) integrated 1.5 h of didactics, 1 h of interactive clinical cases and five “observed” extended basic CCEs (performed by the expert and directly observed by residents). The practical program included five “tutored” extended basic CCEs (performed by the residents under the expert’s tutoring during one-on-one sessions) and at least 5 “offline reviewed” extended basic CCEs (performed independently by the residents and recorded in the ultrasound machine, thereafter reviewed offline by the expert). The time duration for each CCE was recorded prospectively. All CCEs were performed on ICU patients, and care was taken to systematically address all clinical questions covered by extended basic CCE examinations (see Additional file 1).

#### Training program II

Six residents (group II) participated in training program II conducted within the ICU of the University Teaching Hospital of Tenon (Paris, France) between November 2012 and November 2013. This training program differed from program I as follows: (1) a 3-month theoretical and practical appraisal; (2) an extra hour of interactive clinical cases; (3) at least 25 “offline reviewed” extended basic CCEs were required; and (4) the ability to recognize a paradoxical septum was taught (see Additional file 1).

### Evaluation

Both training programs were followed by a 4- and 3-month evaluation period in group I and group II, respectively, to be consistent with the 6-month rotation period of our residents. During this period, all consecutive patients requiring an echocardiography examination at admission or during their ICU stay were studied, unless residents or the expert was not available. Patients with congenital heart disease or surgical cardiac valve replacement or repair were excluded. A recently trained resident and the expert consecutively performed a “paired” extended basic CCE in a random order. There was no alteration in hemodynamic profile and no modification of treatment between the two examinations. Both the resident and the expert had the same information regarding the patient’s medical history and clinical status; both interpreted the “paired” extended basic CCEs online at the bedside. They then independently completed two forms including qualitative, semiquantitative and quantitative clinical questions. At the end of each “paired” extended basic CCE, the expert graded the global imaging quality of his examination as: “high quality,” “moderate quality” or “low quality.” To assess the feasibilities and diagnostic performances of two different training programs, the agreement between the diagnoses raised by the residents and the expert was studied.

### Extended basic critical care transthoracic echocardiography (CCE)

The extended basic CCEs were performed using two recent ultrasound systems equipped with full Doppler capability and a tissue Doppler imaging program (HD11XE system using C4-2 transducer in Colombes ICU; CX50 system using S5-1 transducer in Tenon ICU; Philips Ultrasound, Bothell, WA). The extended basic CCEs aimed to:

- Obtain four cardiac acoustic windows: parasternal short- and long-axis views, apical four-chamber view and subcostal four-chamber view
- Answer the following semiquantitative clinical questions: global LV dysfunction (no, moderate or severe dysfunction); right ventricular (RV) dilatation (no, moderate or severe dilatation); left-sided valvular regurgitation or stenosis (no, mild, significant); pericardial fluid (no fluid, no significant fluid, significant fluid, tamponade); and the respiratory variation of the inferior vena cava (IVC) (no collapsible, collapsible)
- Answer the following qualitative clinical questions: LV contraction pattern (homogeneous, heterogeneous); paradoxical interventricular septum (no, yes; second training program only)
- Answer the following quantitative clinical questions: global LV function [visual evaluation of the LV ejection fraction (LVEF)]; stroke volume (left ventricle outflow tract velocity time integral); LV filling pressure parameters; LV diastolic function [E/A ratio, E/e′ ratio (E = early transmitral velocity; A = late transmitral velocity; e′ = lateral early diastolic velocity of the myocardium at the level of the lateral mitral annulus)]; and aortic valve peak velocity.

Additional details on the methods for answering these clinical questions are provided in Additional file 1. The four cardiac acoustic windows obtained by residents and the expert were saved through an external digital video recording system and analyzed blindly by two independent experienced cardiologists different from the expert. For each cardiac acoustic window obtained, the imaging quality was graded as follows: “optimal” (clear visualization of all anatomical structures) or “not optimal.” The time used for screening was calculated as the time from the start to the end of the examination.

### Statistical analysis

Results are expressed as mean ± standard deviation (SD), or number (percentage), unless otherwise stated. The durations of the CCEs were compared between investigators using a paired Wilcoxon test. Conditions and indications of “paired” CCEs were compared between group I and group II using a Chi-square test. The proportions of “optimal” vs “not optimal” imaging quality and unanswered clinical questions (no response to clinical question) were compared between residents and the expert using the Chi-square McNemar test. In patients for whom the qualitative and semiquantitative clinical questions could be addressed by both resident and expert, the agreement between responses provided by the two investigators was assessed using the Cohen’s κ coefficient and 95 % confidence intervals (CIs) [9]: The kappa value for agreement was interpreted as follows: poor <0.20; fair 0.21–0.40; moderate 0.41–0.60; good 0.61–0.80; and very good 0–81 to 1.0. In patients for whom the quantitative clinical questions could be addressed by both resident and expert, the reproducibility between responses was evaluated using the concordance correlation coefficient (CCC) elaborated by Lin [10]. CCC can vary from −1 to 1, and values above 0.70 are regarded as evidence of good reliability. Cohen’s κ coefficient and CCC were compared between group I and group II using the bootstrap procedure using 1000 resamples [11]. The agreement between quantitative responses was graphically appreciated according to Bland and Altman [12]: Bias (mean difference) ± standard deviation (SD) and the lower and upper 95 % limits of agreement (LoA as bias ± 1.96 SD) were calculated. To determine the numbers of false-positive and false-negative results yielded by residents, the results obtained by the expert were considered as the reference. A *p* value of <0.05 was statistically significant.

## Results

### Training

Details of total training hours of the two programs are shown in Table 1, along with the number of examinations completed during training.

**Table 1:** Two training programs of extended basic critical care transthoracic echocardiography (CCE)

	Group I (*n* = 5)	Group II (*n* = 6)	p
Total program duration, h, mean ± SD	7.1 ± 0.8	17.5 ± 2.0	–
Theoretical program			
Didactics, h	1.5	1.5	–
Interactive clinical cases, h	1.0	2.0	–
Number of “observed” CCEs	5	5	–
Total duration of “observed” CCEs, h, mean ± SD	1.4 ± 0.4	2.1 ± 0.1	0.02
Practice program			
Number of “tutored” CCEs	5	5	–
Duration of “tutored” CCEs, h, mean ± SD	1.8 ± 0.3	2.7 ± 0.3	0.01
Number of “offline reviewed” CCEs, mean (range)	5 (3–5)	22 (18–26)	–
Total duration of “offline reviewed” CCEs, h, mean ± SD	1.4 ± 0.1	9.2 ± 1.6	<0.0001

*SD* standard deviation; *CCE* critical care transthoracic echocardiography

### “Paired” extended basic CCEs

Group I residents performed 136 “paired” extended basic CCEs (mean 27; range 22–32) in 115 patients (62 men; mean ± SD age 64 ± 18 years; Simplified Acute Physiologic Score [SAPS] II 37 ± 18). Group II residents performed 158 “paired” extended basic CCEs (mean 26; range 21–31) in 108 patients (64 men; age 58 ± 18 years; SAPS II 42 ± 22). Indications, conditions and global imaging qualities (graded by the expert) of CCEs were similar between both groups (Table 2).

**Table 2:** Conditions, indications and global imaging qualities (graded by the expert) of “paired” extended basic critical care transthoracic echocardiograms

	Group I (136 CCEs)	Group II (158 CCEs)	*p*
Reason for ICU admission, *n* (%)			
Medical	116 (85)	121 (77)	0.06
Complicated surgery	20 (15)	37 (23)	
Invasive mechanical ventilation, *n* (%)	65 (48)	65 (41)	0.25
Indication for CCE, *n* (%)			
Acute respiratory failure	78 (57)	82 (52)	0.35
Acute circulatory failure	53 (39)	66 (42)	0.63
Acute lung injury/ARDS	43 (32)	35 (22)	0.07
Pulmonary edema	24 (18)	25 (16)	0.68
Combined acute circulatory and respiratory failures	21 (15)	28 (18)	0.60
Exacerbation of chronic respiratory failure	12 (9)	13 (8)	0.86
Suspected/diagnosed endocarditis	8 (6)	18 (11)	0.10
Suspected/diagnosed pericarditis/myocarditis	6 (4)	10 (6)	0.50
Acute coronary syndrome	6 (4)	10 (6)	0.47
Suspected/diagnosed pulmonary embolism	5 (4)	6 (4)	0.96
Acute chest syndrome in sickle cell disease	0	10 (6)	0.003
Post-cardiac arrest	1 (1)	7 (4)	0.07
Weaning failure from ventilator	4 (3)	2 (1)	0.42
Global imaging quality of CCE, *n* (%)			
High	82 (60)	92 (62)	0.90
Moderate	30 (22)	31 (21)	
Low	21 (15)	26 (17)	
Unknown	3 (2)	9 (7)	

*CCE* critical care transthoracic echocardiography

When compared to the expert, group I and group II residents performed longer extended basic CCEs (22 ± 8 vs 12 ± 6 min, *p* < 0.0001; 22 ± 10 vs 13 ± 7 min, *p* < 0.0001, respectively) with less optimal acoustic windows (1.8 ± 1.2 vs 2.8 ± 1.1, *p* < 0.0001; 2.5 ± 1.2 vs 3.1 ± 1.2, *p* < 0.0001, respectively). Group I residents recorded at least one optimal acoustic window in 105 of 127 (83 %) extended basic CCEs versus 124 of 127 (98 %) extended basic CCEs by the expert (*p* < 0.0001). Group II residents recorded at least one optimal acoustic window in 112 of 123 (91 %) extended basic CCEs versus 116 of 123 (94 %) extended basic CCEs by the expert (*p* < 0.33).

### Diagnostic performance

While residents in group I yielded significantly more unanswered clinical questions than the expert (264 [13 %] vs 135 [7 %] of 2040 clinical questions; *p* < 0.0001), the number of unanswered clinical questions was not statistically different between residents in group II and the expert (respectively 235 [9 %] vs 218 [8.5 %] of 2528 clinical questions; *p* = 0.21) **(**Table 3).

**Table 3:** Number of unanswered clinical questions by residents and the expert

Clinical questions	Unanswered questions by residents/expert, n	
	Group I (136 CCEs)	Group II (158 CCEs)
Global LV systolic function	0/0	2/1
Heterogeneous LV contraction	4/6	6/20
RV dilatation	1/2	6/1
Paradoxal septum	–	6/4
Significant LS valve disease	17/4	12/5
Significant MR	8/2	10/5
Significant MS	20/1	10/5
Significant AR	8/1	15/4
Significant AS	24/2	14/3
Pericardial effusion	2/1	1/0
Significant respiratory variations of IVC diameter	50/28	35/20
LVEF	2/1	4/1
LVOT VTI	37/15	15/31
LV filling pressure and diastolic function^a^		
Ratio E/A	20/23	41/49
Ratio E/e′	13/26	29/48
Aortic peak velocity	58/23	29/21

*A* late transmitral velocity, *AR* aortic regurgitation, *AS* aortic stenosis, *CCE* critical care transthoracic echocardiography *CI* confidence interval, *E* early transmitral velocity, *e′* lateral early diastolic velocity of the myocardium at the level of the lateral mitral annulus, *IVC* inferior vena cava, *LS* left-sided, *LV* left ventricular, *LVEF* left ventricular ejection fraction, *LVOT* left ventricular outlfow tract, *MR* mitral regurgitation, *MS* mitral stenosis, *RV* right ventricular, *VTI* velocity time integral

^a^Sinus rhythm

### Semiquantitative and qualitative clinical questions

Table 4 presents the answers to semiquantitative and qualitative clinical questions by residents compared with the expert as a reference. When addressed by the expert and the residents, the classification of global LV systolic function, and pericardial effusion appraised by group I and group II residents showed very good agreement with the expert. Group I and group II residents adequately qualified global LV systolic function as normal or not in 120/136 (88 %) evaluated patients (κ 0.75, 95 % CI 0.64–0.86) and in 140 of 156 (90 %) evaluated patients (κ 0.77, 95 % CI 0.66–0.88), respectively. Both groups assessed the degree to which LV systolic function was depressed with a good and very good agreement (group I: κ 0.69, 95 % CI 0.60–0.79; group II: κ 0.82, 95 % CI 0.73–0.91; full details can be found in Additional file 2). The two cases of tamponade were both detected by the residents in group II. Compared with group I residents, group II residents agreements were good and higher for the assessing of the degree to which RV was dilated (κ 0.71, 95 % CI 0.50–0.77 vs κ 0.51, 95 % CI 0.24–0.61, *p* = 0.07; Additional file 3), the detection of a severe dilated RV (κ 0.80, 95 % CI 0.56–0.96 vs κ 0.64, 95 % CI 0.38–0.91, *p* = 0.35) and the detection of a significant left-sided valve disease (κ 0.79, 95 % CI 0.66–0.93 vs κ 0.54, 95 % CI 0.33–0.75, *p* = 0.02). Group II residents detected a paradoxical septum with a moderate agreement (κ 0.65, 95 % CI 0.37–0.93). Lastly, both groups detected a heterogeneous LV contraction and a significant respiratory variation of IVC diameter with a fair to moderate agreement.

**Table 4:** Answers to semiquantitative and qualitative clinical questions by residents and the expert

Semiquantitative and qualitative clinical questions^a^	Resident accuracy, % Sens/Spe/PPV/NPV		Expert’s positive results, n (%)		Agreement between residents and expert, κ (95 % CI)		
	Group I	Group II	Group I	Group II	Group I	Group II	p*
Global LV systolic function (n = 136/ n = 156)							
Any dysfunction	96/85/75/97	93/92/79/97	45 (33)	42 (26)	0.75 (0.64–0.86)	0.77 (0.66–0.88)	0.93
Severe dysfunction	58/96/69/93	93/99/93/99	19 (14)	15 (9)	0.57 (0.37–0.78)	0.93 (0.82–1)	0.004
Heterogeneous LV contraction (n = 115/n = 136)	65/89/71/85	19/80/50/49	34 (30)	41 (30)	0.55 (0.38–0.72)	0.49 (0.33–0.65)	0.54
RV dilatation (n = 133/n = 152)							
Any dilatation	56/90/56/90	80/89/73/93	25 (19)	40 (26)	0.46 (0.27–0.65)	0.67 (0.54–0.80)	0.08
Severe dilatation	55/99/86/96	88/98/70/99	11 (8)	8 (5)	0.64 (0.38–0.91)	0.80 (0.56–0.96)	0.35
Paradoxical septum (−/n = 150)	–	50/100/100/97	–	10 (7)	–	0.65 (0.37–0.93)	–
Significant LS valve disease (n = 119/n = 146)	55/95/69/91	91/95/76/98	16 (13)	21 (15)	0.54 (0.33–0.75)	0.79 (0.66–0.93)	0.02
Significant MR (n = 127/n = 145)	50//98/40/98	80/96/57/99	4 (3)	10 (7)	0.42 (0.01–0.84)	0.64 (0.40–0.87)	0.42
Significant MS (n = 116/n = 145)	50/100/100/98	67/100/100/99	4 (3)	3 (2)	0.66 (0.22–1)	0.80 (0.41–1)	0.41
Significant AR (n = 128/n = 141)	0/98/0/98	100/100/100/100	3 (2)	2 (2)	–0.02 (–0.04–0)	1	0.001
Significant AS (n = 112/n = 143)	60/100/100/98	86/100/100/99	5 (4)	7 (5)	0.74 (0.40–1)	0.92 (0.76–1)	0.37
Pericardial effusion (n = 133/n = 157)							
Any	87/94/62/99	93/96/68/99	13 (8)	14 (9)	0.83 (0.67–0.99)	0.76 (0.60–0.93)	0.59
Significant	100/100/100/100	100/100/100/100	8 (6)	7 (4)	1	1	–
Tamponade	–	100/100/100/100	0	2 (1)	–	1	–
Significant respiratory variations of IVC diameter (n = 76/n = 116)	53/95/75/88	42/84/59/72	17 (22)	41 (35)	0.53 (0.30–0.77)	0.27 (0.09–0.45)	0.09

*AR* aortic regurgitation, *AS* aortic stenosis, *CI* confidence interva, *LV* left ventricular, *IVC* inferior vena cava, *LS* left-sided, *MR* mitral regurgitation, *MS* mitral stenosis, *NPV* negative predictive value, *PPV* positive predictive value, *RV* right ventricular

* Comparison of κ values between group I and group II

^**a**^The number of comparisons is noted for each clinical question and for each group (group I/group II)

### Quantitative clinical questions using Doppler capabilities

**Fig. 1:**
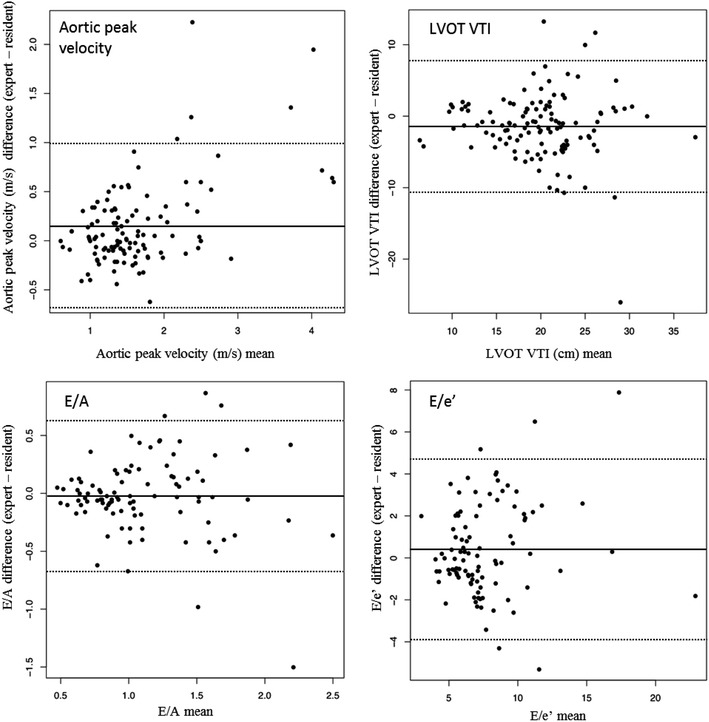
Bland–Altman plot between quantitative questions using Doppler capabilities provided by group II residents and the expert for the whole set of pairs of measurements. A, late transmitral velocity; E, early transmitral velocity; e′, lateral early diastolic velocity of the myocardium at the level of the lateral mitral annulus; LVEF, left ventricular ejection fraction; LVOT, left ventricular outflow tract; VTI, velocity time integral. *Dotted line*, mean difference (bias); *solid lines*, limits of agreement (bias ± 1.96 SD)

Table 5 presents the CCCs, the bias and the standard deviation (SD) for each quantitative answer provided by residents and the expert as a reference. Except for LVOT VTI, reproducibility between group II residents and the expert was good and significantly higher than between group I residents and the expert regarding aortic peak velocity (CCC 0.82 vs CCC 0.43, *p* = 0.01), E/A ratio (CCC 0.73 vs CCC 0.38, *p* = 0.01) and E/e′ ratio (CCC 0.75 vs CCC 0.53, *p* = 0.049). The Bland–Altman plots demonstrating the bias (95 % LoA) of quantitative Doppler measurements between residents in group II and the expert are shown in Fig. 1. Aortic peak velocity, LVOT VTI, E/A ratio and E/e′ ratio were measured with a very little bias but with wide levels of agreement [respectively, 0.1 m/s (−0.7–1), −1.4 cm (−10.6 to 7.8), 0 (−1 to 0.8) and 0.4 (−3.9 to 4.7)].

**Table 5:** Answers to quantitative clinical questions by residents and the expert

Quantitative questions^a^	CCC (95 % CI)			Bias ± SD	
	Group I	Group II	p*	Group I	Group II
LVEF (%) (n = 133/n = 154)	0.78 (0.70–0.83)	0.82 (0.77–0.87)	0.34	3.1 ± 9	1.3 ± 8.8
LVOT VTI (cm) (n = 92/n = 120)	0.61 (0.48–0.71)	0.65 (0.54–0.74)	0.65	−2.1 ± 4.8	−1.4 ± 4.7
Ratio E/A (n = 87/n = 95)^b^	0.38 (0.20–0.54)	0.73 (0.62–0.81)	0.01	−0.1 ± 0.4	0 ± 0.3
Ratio E/e′ (n = 104/n = 98)^b^	0.53 (0.38–0.65)	0.75 (0.65–0.83)	0.049	0.8 ± 3.4	0.4 ± 2.2
Aortic peak velocity (m/s) (n = 73/n = 117)	0.43 (0.25–0.68)	0.82 (0.76–0.86)	0.01	0.1 ± 0.5	0.1 ± 04

*A* late transmitral velocity, *E* early transmitral velocity, *e′* lateral early diastolic velocity of the myocardium at the level of the lateral mitral annulus, *CCC* concordance correlation coefficient, *LVEF* left ventricular ejection fraction, *LVOT* left ventricular outflow tract, *SD* standard deviation, *VTI* velocity time integral

* Comparison of CCC values between group I and group II

^a^The number of comparisons is noted for each quantitative clinical question and for each group: group I/group II

^b^Sinus rhythm

## Discussion

This is the first study that aimed to validate whether a focused training program designed to achieve essential competences in extended basic CCE including Doppler capabilities for residents with no prior ultrasound experience was adequate. Basic critical care echocardiography by physicians inexperienced in ultrasound has been previously investigated, using training programs of 6–15 h [4–7]. Of note, these programs did not include the use of Doppler and the training objectives were limited to a 2D approach that may considerably narrow the scope of the examination [4–6]. The scanning times of our trainees was 10–15 min longer than these earlier series, related to more assessments using Doppler examination, which goes beyond the scope of basic critical care echocardiography. Even if our longer training program allows acquiring some essential competences for establishing diagnoses and guiding the management of critically ill patients, it seems to be insufficiently to perform a complete extended basic critical care transthoracic echocardiography.

The shorter training program blending approximately 4-h theory and 3-h practical allowed residents to acquire only two important skills using 2D imaging including global systolic LV function and pericardial effusion assessment. However, residents failed to achieve others skills using 2D imaging and Doppler capabilities. The longer training program blending approximately 4 h theory and 12 h practical allowed residents to acquire to a good or very good degree of some essential competences in extended basic CCE, using 2D imaging (global systolic LV function and pericardial effusion assessment, RV size evaluation) and Doppler capabilities (detection of significant left-sided valve including aortic stenosis, mitral stenosis, aortic regurgitation and mitral regurgitation). As expected, LV filling pressure and diastolic function were more adequately assessed by residents trained by the long program than by those trained with the short program, with a good reproducibility but a moderate agreement. Lastly, some skills such as the measurement of the stroke volume, the detection of heterogeneous LV contraction and of paradoxical septum and the assessment of respiratory variation of IVC diameter probably require more in-depth training.

For the first time, our study shows that a focused training program in ICU setting allows to reach competences using Doppler capabilities. Using color and continuous Doppler, the residents trained by the long program adequately detected significant left-sided valve disease, as only 6 % of the evaluated patients were misclassified. Consistent with our results, Mjølstad et al. [13] found that residents undergoing a training program with 4-h theoretical and 95 echocardiograms adequately detected moderate aortic or mitral valve diseases (and missed no severe ones) using a pocket-size handheld echocardiography machine in a non-ICU setting. Conversely to the valve diseases, LV filling pressures and diastolic function assessments using pulsed and tissue Doppler were more challenging, even for the expert in 23 % of the patients, because of no sinus rhythm, tachycardia or low imaging quality. Despite a good reproducibility for the E/A and E/e′ Doppler indices on patients with normal sinus rhythm between the residents (trained with the long program) and the expert, the limits of agreement (bias ± 1.96, SD −0.7 to 0.6 and −4 to 5, respectively) are too large for us to be confident that the measure of residents can be used for clinical purposes. Our results are in the same range as in previous study that reported a moderate interobserver agreement for E/e′ ratio in patients with septic shock, possibly reflecting the difficulty in assessing diastolic function in critically ill patient [14]. Since we found considerable interobserver variability between residents and the expert in stroke volume measurement requiring aortic pulsed-flow Doppler and detailed 2D imaging, our results imply the need for additional training in using these to guide management in critically ill patients.

Some essential basic skills using only 2D imaging seem easier to achieve since, in keeping with recent series [4–7], both groups of residents adequately evaluated global LV systolic function and identified pericardial effusion. Residents trained by the longer training program accurately diagnosed the two cases of tamponade encountered in the current study. Our results indicate that RV size may be more difficult to evaluate, especially when the dilatation RV is moderate, requiring the longer training program. Similarly, DeCara et al. [15] noted substantial differences in the performances of handheld devices to detect RV dysfunction when used by physicians with limited training, compared with an expert in echocardiography. However, some competences using 2D imaging probably require more training, as previously reported [6, 13, 15]. According to our results, Vignon et al. [6] showed that residents who underwent a 12-h training program tended to underestimate respiratory variations of IVC size. In the same way, Mjølstad et al. [13] reported that recently trained residents inadequately assessed the inferior vena cava diameter in a non-ICU setting. Although the visual detection of regional wall motion abnormalities is defined as a basic competence in critical care echocardiography, no previous study in ICU setting has validated the level of training required for its acquisition. Similar to our results, previous studies in a non-ICU setting find that acquisition of this skill by physicians with limited training was particularly difficult [13, 15]. Assessing thickening of every LV walls requires a very good quality of imaging and probably a more important level of training. Lastly, our results confirm the consensus statement on competence in critical care ultrasonography which indicates that accurate identification of paradoxical septal motion may be challenging [1].

Compared to residents trained by the short program, the agreement between responses provided by residents trained by the long program and the expert was generally higher. This may be related to the longer training curriculum, which included more interactive clinical cases, but may also be related to a learning curve effect, since residents trained by the long program performed a mean of 27 examinations, compared with 10 in the short training program. Our learning rate estimates are consistent with the “European Society of Intensive Care Medicine” recommendations of approximately 30 TTEs in ICU patients to reach competence in basic critical care echocardiography [2]. Despite scarce data, experts in echocardiography in the ICU have suggested that “a minimum of 100 full TTEs is required as part of training in advanced critical care transthoracic echocardiography” [3]. It is clear that mastering the technique of advanced level requires a considerable investment in training [16]. However, our study indicates that some essential competences at an advanced critical care transthoracic echocardiography level were achieved with approximately 30 extended basic critical care transthoracic echocardiography. One strength is that the training program required trainees to scan various ICU patients with and without mechanical ventilation, which provided a rich source of practical experience. Lastly, apart from the time taken by the expert to teach, we did not invest in additional training materials or echocardiography simulators. As such, others may readily replicate our program.

Our study has some limitations. First, the training programs were conducted in two different places, at different times. However, indications, conditions and global imaging qualities of “paired” extended basic CCEs were mainly similar between both study centers, and both trainings were conducted with non-cardiologist residents with no prior experience in ultrasound. Second, we have included a limited number of trainees because of our small number of residents, their 6-month rotation period and the exclusion of those who had experience in ultrasound. However, each recently formed resident performed a large number of CCE during the evaluation period. Third, the mean duration of extended basic CCE examination was very long, even after a larger number of “offline reviewed” extended basic CCEs. We consider this point to be crucial because hemodynamic evaluation has to be performed quickly in order to rapidly optimize treatment. Fourth, because of the workload in the ICU, the residents were often hard-pressed to find time to perform the CCEs. A rotation specifically dedicated to learning echocardiography might be one way to improve results substantially. Fifth, our study wasn’t designed to assess the learning curve of residents. Finally, because the simulation system was unavailable in our units, this very efficient learning modality was not been tested in the study.

## Conclusions

A focused training program appears satisfactory to teach residents to adequately address some important clinical questions for a comprehensive hemodynamic assessment in critical care patients. However, a complete extended basic critical care transthoracic echocardiography including common parameters derived from spectral Doppler and tissue Doppler imaging requires a larger training program. Future research should aim to assess the ability of these training programs to result in adequate therapeutic proposals.

## Additional files

10.1186/s13613-016-0150-8 Theoretical curriculum for echocardiographic patterns related to clinical questions addressed by extended basic critical care transthoracic echocardiography.
10.1186/s13613-016-0150-8 Assessment of global left ventricular systolic function by residents in group I (*n* = 136) (top table) and by residents in group II (n = 156) (bottom table).
10.1186/s13613-016-0150-8 Assessment of right ventricular size by residents in group I (*n* = 133) (top table) and by residents in group II (n =152) (bottom table).

## Abbreviations

CCC: concordance correlation coefficient
CCE: critical care transthoracic echocardiography
CIs: confidence intervals
LV: left ventricular
LVEF: left ventricular ejection fraction
LVOT: left ventricular outflow tract
ICU: intensive care unit
IVC: inferior vena cava
RV: right ventricular
SAPS: Simplified Acute Physiologic Score
SD: standard deviation
VTI: velocity time integral
