# Supplementary material for: Can we improve transthoracic echocardiography training in non-cardiologist residents? Experience of two training programs in the intensive care unit
Source: Ann Intensive Care. 2016 May 17;6:44. doi: 10.1186/s13613-016-0150-8 (PMC4870482; doi:10.1186/s13613-016-0150-8)
Supplement: Supplementary file 1 — 10.1186/s13613-016-0150-8 Theoretical curriculum for echocardiographic patterns related to clinical questions addressed by extended basic critical care transthoracic echocardiography. [file 13613_2016_150_MOESM1_ESM.doc]

**Additional file 1.** Theoretical curriculum for echocardiographic patterns related to clinical questions addressed by extended basic critical care transthoracic echocardiography (CCE)

**Didactic Curriculum**

- Ultrasound basics and image optimization
- Doppler basics (color, pulsed, continuous, and tissue)
- Standard transthoracic views of the heart with normal cardiac anatomy: parasternal long- and short-axis views, apical four-chamber view, subcostal four-chamber view, and inferior vena cava (IVC)
- Echocardiographic patterns related to semi-quantitative clinical questions addressed by extended basic CCE:
- Global left ventricular (LV) systolic function using two-dimensional (2D) imaging: normal, moderately depressed (visual semi-quantitative evaluation of LV ejection fraction [LVEF] 30–55%), or severely depressed (visual semi-quantitative evaluation of LVEF < 30%) [1]
- Global right ventricular (RV) size using 2D imaging: normal, moderately dilated (ratio of RV/LV end-diastolic area measured in the apical four-chamber view > 0.6), or severely dilated (ratio of RV/LV end-diastolic area measured in the apical four-chamber view > 1) [2]
- Mitral regurgitation (MR) using 2D imaging and basic color Doppler: normal, mild regurgitation (no significant systolic mitral jet), or significant regurgitation (prominent mitral flail leaflet or MR jet occupying > 20% of the left atrium area) [3]
- Aortic regurgitation (AR) using 2D imaging and basic color Doppler: normal, mild regurgitation (no significant diastolic aortic jet), or significant regurgitation (prominent aortic flail leaflet or AR jet occupying > 20% of the LV area and/or extending into the LV apex [4]
- Aortic valvular stenosis using 2D imaging and transaortic continuous Doppler: normal, mild stenosis (moderate calcified and/or restricted aortic cusps and aortic valve peak velocity > 2 m/s) or significant stenosis (severe calcified and/or restricted aortic cusps and aortic valve peak velocity > 3 m/s and/or transaortic mean gradient > 25 mmHg) [5,6]
- Mitral valvular stenosis using 2D imaging and transmitral continuous Doppler: normal, mild stenosis (moderate calcified and/or restricted mitral cusps and transmitral mean gradient < 5 mmHg) or significant stenosis (severe calcified and/or restricted mitral cusps and transmitral mean gradient ≥ 5 mmHg) [5]
- Significant left-sided valve disease: significant MR, AR, aortic stenosis (AS), or mitral stenosis (MS)
- Pericardial fluid using 2D imaging: none, non-significant (pericardial effusion localized, and assessed < 5 mm), significant (circumferential pericardial effusion or assessed > 5 mm), tamponade [7] (significant pericardial effusion, collapsed [right] cardiac cavities and dilated noncollapsible IVC)
- Respiratory variation of IVC diameter using 2D imaging: collapsible vessel (visually assessed diameter reduction > 50% during inspiration with spontaneous respiratory effort or during expiration on ventilatory support without spontaneous respiratory) or noncollapsible vessel (absence or nonsignificant respiratory variations of IVC diameter) [8]
- Echocardiographic patterns related to quantitative clinical questions addressed by extended basic CCE:
- Global LV systolic function using 2D imaging: visual quantitative evaluation of LVEF
- Quantitative assessment of LV volume (second training program only) using the monoplane Simpson’s method to measure the telediastolic LV volume in the apical chamber view. LV dilation corresponded to a telediastolic LV volume > 75 ml indexed to body surface area [4]
- Parameters of the stroke volume: LVOT velocity time integral calculated by tracing the envelope of the flow with pulsed Doppler placed at the level of the LVOT
- Parameters of LV filling pressures and the LV diastolic function using the pulsed-wave Doppler recorded at the tip of the mitral valve in the apical four-chamber view (early [E] and late [A] transmitral velocities) and tissue Doppler recorded at the lateral corner of the mitral annulus in the four-chamber view (lateral early diastolic velocity of the myocardium at the level of the lateral mitral annulus [e']): measurement of E/A mitral ratio and E/e' mitral ratio [9]
- Aortic valve peak velocity measured with continuous Doppler placed across the aortic valve
- Echocardiographic patterns related to qualitative clinical questions addressed by extended basic CCE:
- Homogeneous/heterogeneous LV contraction pattern using 2D imaging: identification of LV regional wall motion abnormalities as a heterogeneous pattern of systolic LV wall thickening (precise identification of segmental distribution not required)
- Recognition of a paradoxical septum (second training program only) using 2D imaging in the parasternal short-axis view [10]: abnormal curvature and systolic recruitment toward RV ejection.

**Interactive Clinical Cases**

Syndromes covered by extended basic CCE:

- Clinical syndromes covered by basic critical care echocardiography as previously described [7]
- Clinical syndromes requiring some components of competences in advanced critical care echocardiography [7]: LV systolic failure with low cardiac index; LV diastolic failure; elevation of LV filling pressures measured in spontaneously breathing patients; significant native left-sided valve disease using color and spectral Doppler.

**Observed extended basic CCE**

- Operating information and machine settings
- Probe positioning and orientation, normal views, identification of normal anatomical structures and landmarks
- Validation of 2D and Doppler measurements (electronic calipers) used in the study.

**REFERENCES**

1. Vignon P, Mücke F, Bellec F, Marin B, Croce J, Brouqui T, et al. Basic critical care echocardiography: validation of a curriculum dedicated to noncardiologist residents. Crit. Care Med. 2011;39:636–42.

2. Kasper W, Meinertz T, Kersting F, Löllgen H, Limbourg P, Just H. Echocardiography in assessing acute pulmonary hypertension due to pulmonary embolism. Am. J. Cardiol. 1980;45:567–72.

3. Helmcke F, Nanda NC, Hsiung MC, Soto B, Adey CK, Goyal RG, et al. Color Doppler assessment of mitral regurgitation with orthogonal planes. Circulation. 1987;75:175–83.

4. Feigenbaum H, Armstrong W, Ryan T. Feigenbaum’s echocardiography. 6th ed. Philadelphia, Pennsylvania: Lippincott Williams & Wilkins; 2004.

5. American College of Cardiology/American Heart Association Task Force on Practice Guidelines, Society of Cardiovascular Anesthesiologists, Society for Cardiovascular Angiography and Interventions, Society of Thoracic Surgeons, Bonow RO, Carabello BA, et al. ACC/AHA 2006 guidelines for the management of patients with valvular heart disease: a report of the American College of Cardiology/American Heart Association Task Force on Practice Guidelines (writing committee to revise the 1998 Guidelines for the Management of Patients With Valvular Heart Disease): developed in collaboration with the Society of Cardiovascular Anesthesiologists: endorsed by the Society for Cardiovascular Angiography and Interventions and the Society of Thoracic Surgeons. Circulation. 2006;114:e84–231.

6. Oh J, Seward J, Tajik A. The Echo Manual. 3rd ed. Philadelphia, Pennsylvania: Lippincott Williams & Wilkins; 1999.

7. Mayo PH, Beaulieu Y, Doelken P, Feller-Kopman D, Harrod C, Kaplan A, et al. American College of Chest Physicians/La Societe de Reanimation de Langue Francaise statement on competence in critical care ultrasonography. Chest. 2009;135:1050–60.

8. Kircher BJ, Himelman RB, Schiller NB. Noninvasive estimation of right atrial pressure from the inspiratory collapse of the inferior vena cava. Am. J. Cardiol. 1990;66:493–6.

9. Nagueh SF, Middleton KJ, Kopelen HA, Zoghbi WA, Quinones MA. Doppler tissue imaging: a noninvasive technique for evaluation of left ventricular relaxation and estimation of filling pressures. J Am Coll Cardiol. 1997;30:1527–33.

10. Jardin F, Dubourg O, Bourdarias JP. Echocardiographic pattern of acute cor pulmonale. Chest. 1997;111:209–17.
