# Supplementary material for: Can we improve transthoracic echocardiography training in non-cardiologist residents? Experience of two training programs in the intensive care unit
Source: Ann Intensive Care. 2016 May 17;6:44. doi: 10.1186/s13613-016-0150-8 (PMC4870482; doi:10.1186/s13613-016-0150-8)
Supplement: Supplementary file 2 — 10.1186/s13613-016-0150-8 Assessment of global left ventricular systolic function by residents in group I (n = 136) (top table) and by residents in group II (n = 156) (bottom table). [file 13613_2016_150_MOESM2_ESM.docx]

**Additional file 2.** Assessment of global left ventricular systolic function by residents in group I (n = 136) (top table) and by residents in group II (n = 156) (bottom table). doc

| **Global left ventricular systolic function** | | **Assessment by residents** | | |
| --- | --- | --- | --- | --- |
|  |  | Normal | Moderately depressed | Severely depressed |
| **Assessment by expert** | Normal | 77 | 14 | 0 |
|  | Moderately depressed | 2 | 19 | 5 |
|  | Severely depressed | 0 | 8 | 11 |

κ, 0.69 95%CI 0.60–0.79.

| **Global left ventricular systolic function** | | **Assessment by residents** | | |
| --- | --- | --- | --- | --- |
|  |  | Normal | Moderately depressed | Severely depressed |
| **Assessment by expert** | Normal | 102 | 11 | 1 |
|  | Moderately depressed | 3 | 24 | 0 |
|  | Severely depressed | 0 | 1 | 14 |

κ, 0.82 95%CI 0.73–0.91.

CI = confidence interval.
