# Supplementary material for: Can we improve transthoracic echocardiography training in non-cardiologist residents? Experience of two training programs in the intensive care unit
Source: Ann Intensive Care. 2016 May 17;6:44. doi: 10.1186/s13613-016-0150-8 (PMC4870482; doi:10.1186/s13613-016-0150-8)
Supplement: Supplementary file 3 — 10.1186/s13613-016-0150-8 Assessment of right ventricular size by residents in group I (n = 133) (top table) and by residents in group II (n =152) (bottom table). [file 13613_2016_150_MOESM3_ESM.doc]

**Additional file 3.** Assessment of right ventricular size by residents in group I (*n* = 133) (top table) and by residents in group II (n = 152) (bottom table). doc

| **Right ventricular size** | | **Assessment by residents** | | |
| --- | --- | --- | --- | --- |
|  |  | Not dilated | Moderately dilated | Severely dilated |
| **Assessment by expert** | Not dilated | 97 | 10 | 1 |
|  | Moderately dilated | 8 | 6 | 0 |
|  | Severely dilated | 3 | 2 | 6 |

κ, 0.51 95%CI 0.24–0.61

| **Right ventricular size** | | **Assessment by residents** | | |
| --- | --- | --- | --- | --- |
|  |  | Not dilated | Moderately dilated | Severely dilated |
| **Assessment by expert** | Not dilated | 100 | 11 | 1 |
|  | Moderately dilated | 8 | 22 | 2 |
|  | Severely dilated | 0 | 1 | 7 |

κ, 0.71 95%CI 0.50–0.77

CI = confidence interval.
